# Semiconducting Carbon Nanotube-Based Nanodevices for Monitoring the Effects of Chlorphenamine on the Activities of Intracellular Ca^2+^ Stores

**DOI:** 10.1155/2022/9019262

**Published:** 2022-03-02

**Authors:** Viet Anh Pham Ba, Ngoc Pham Van Bach, Thien Nguyen Luong, Khoa Viet Nguyen

**Affiliations:** ^1^Graduate University of Science and Technology, Vietnam Academy of Science and Technology, Hanoi, Vietnam; ^2^Department of Environmental Toxicology and Monitoring, Hanoi University of Natural Resources and Environment, Hanoi, Vietnam; ^3^Space Technology Institute, Vietnam Academy of Science and Technology, Hanoi, Vietnam; ^4^Institute of Mechanics, Vietnam Academy of Science and Technology, Hanoi, Vietnam

## Abstract

We report a flexible and noninvasive method based on field-effect transistors hybridizing semiconducting single-walled carbon nanotubes for monitoring the effects of histamine on Ca^2+^ release from the intracellular stores of a nonexcitable cell. These nanodevices allowed us to evaluate the real-time electrophysiological activities of HeLa cells under the stimulation of histamine via the recording of the conductance changes of the devices. These changes resulted from the binding of histamine to its receptor type 1 on the HeLa cell membrane. Moreover, the effects of chlorphenamine, an antihistamine, on the electrophysiological activities of a single HeLa cell were also evaluated, indicating that the pretreatment of the cell with chlorpheniramine decreased intracellular Ca^2+^ release. Significantly, we only utilized a single nanodevice to perform the measurements for multiple cells pretreated with various concentrations of chlorphenamine. This enabled the statistically meaningful analysis of drug effects on cells without errors from device variations. Obtained results indicated the novel advantages of our method such as real-time monitoring and quantitative capability. Our devices, therefore, can be efficient tools for biomedical applications such as electrophysiology research and drug screening.

## 1. Introduction 

Intracellular Ca^2+^ stores have been verified to be associated with many electrophysiological activities in various cell lines, including HeLa cells. Several possible mechanisms of Ca^2+^ release from intracellular stores have been discovered. The intracellular Ca^2+^ release regulated by inositol 1, 4, 5-triphosphates (IP_3_) is a main mechanism among them, which plays an important function in cellular reactions relating to various diseases [1–3]. For example, the activation of histamine H1 receptors (H1Rs) embedded in the plasma membrane of HeLa cells leads to the formation of cytosolic IP_3_. Subsequently, IP_3_ binds to its receptors, which acts as the Ca^2+^ channels of intracellular Ca^2+^ stores [4–6]. This results in the increase of cytosolic-free calcium concentrations, which drives the depolarization of the cell membrane as well as the alteration of the cell membrane potential [7]. Based on the monitoring of the membrane potential change, the histamine effects on the activities of the Ca^2+^ stores could be evaluated. To monitor the electrophysiological effects on the cells, detection tools need to be sensitive and reliable.

Conventional optical imaging methods, which have been applied to monitor the changes of intracellular Ca^2+^, include radioactive isotopic analyses and fluorescent assays [8–11]. Isotopic labeling assays allow us to trace analytes since these assays do not have any influence on the structure or reactivity of the analytes [12]. However, radioactive agents may cause hazards to cell survival and waste issues. Although fluorescent assays have high sensitivity and specificity, this method requires time-consuming preparation procedures [13]. Moreover, the photo-signals of both radioactive isotopes and fluorophores are not stable for a long time [12, 13]. Besides, electrophysiological measurement methods such as patch-clamp techniques have shown distinctive features for the detection of bioelectrical signals [14, 15]. However, the patch-clamp techniques still may need a cellular invasion and complex manipulation. In addition, these techniques just measure the local signals of ion flows across a cell membrane [16].

Recently, the prompt development of nanomaterials science has facilitated the application of nanostructure devices in biomedical research [17, 18]. These nanodevices have been, therefore, widely utilized to explore the electrophysiological activities of adherent cells grown on the devices with simple and easy procedures [19–21]. For example, since nanodevices hybridizing with carbon nanotubes (CNTs) were proven to be appropriate devices for living cell studies, CNT-field effect transistors (FETs) have been applied to monitor the activities of various cells [22–26]. Nonetheless, the growth of cells directly on the device can affect the characterization of the device and result in difficulty when comparing and estimating results among measurements.

Herein, we developed a method for the activity monitoring of intracellular Ca^2+^ stores using semiconducting CNT-based nanodevices (SCN devices). In our work, a HeLa cell was placed on an SCN device via a microcapillary for the monitoring of its electrophysiological responses to histamine. Using this method, we could estimate the effects of histamine on Ca^2+^ release from the intracellular stores of HeLa cells. Significantly, the measurements could be repeated with other cells using the same device, enabling quantitative evaluation without suffering from the characteristic variation of different devices. The electrophysiological responses of the cells to histamine were attributed to the depolarization of the cell membranes caused by the histamine stimulation of H1Rs embedded in the cell membranes [6, 27]. Also, the effects of chlorphenamine, an antihistamine, on the histamine-induced activities of the Ca^2+^ stores were evaluated by using only a SCN device. The results indicate that histamine-induced Ca^2+^ release from the stores could be reduced by the pretreatment of cells with chlorphenamine in a dose-dependent manner. This means chlorphenamine could partially prevent the stimulation of H1Rs in HeLa cells via histamine as in previous reports [28, 29]. Importantly, this work may supply a simple but efficient method for the monitoring of electrophysiological activities at a single-cell level, which should suggest various applications of biosensor technology in biomedical research.

## 2. Materials and Methods

### 2.1. Materials

Semiconducting single-walled carbon nanotubes (ssCNTs), histamine, chlorphenamine, and other chemical reagents were purchased from Sigma-Aldrich and used as received. The ssCNTs had a diameter of 0.7–1.1 nm and a length of 300–2300 nm.

HeLa cells were provided by the University of Science and Technology of Hanoi (USTH) (Vietnam). The HeLa cells were grown on a culture Petri dish in Dulbecco's Modified Eagle Medium supplemented with fetal bovine serum (10% v/v), L-glutamine (2 mM), penicillin (100 U/mL), and streptomycin (100 *µ*g/mL) at 37°C in the atmosphere of 5% CO_2_.

### 2.2. Fabrication of Nanodevices

**Figure 1:**
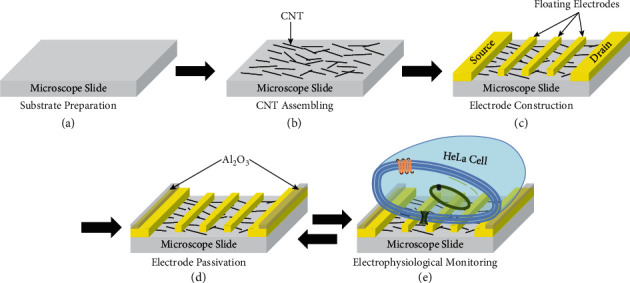
Schematic diagram sketching the fabrication procedures of a SCN device and the electrophysiological monitoring of a single HeLa cell using the device. (a) Preparation of a clean microscope slide. (b) Assembling of aligned ssCNTs on the clean slide. (c) Construction of the source, drain, and floating electrodes on the CNT network. (d) Passivation of the source and drain electrodes by an Al_2_O_3_ layer. (e) Loading and monitoring of a HeLa cell on the SCN device. The drawing is not to scale.

The experimental procedures for the real-time monitoring of the electrophysiological responses of HeLa cells by using SCN devices are illustrated in Figure 1. The fabrication process of the SCN devices was performed as in previous reports [30, 31]. Firstly, microscope slides (Marienfeld, Germany) with the size of 24 mm width × 60 mm length × 0.17 mm thickness were immersed into a piranha solution to create flat and clean substrates (Figure 1(a)). In the next step, aligned CNTs were absorbed on the substrates via a spin-coating method (Figure 1(b)). Since the treated slide substrate is flat and clean, CNT can easily attach to the substrate. Moreover, the spin-coating method can increase the assembly of CNTs on the substrates towards the same direction. Then, source, drain, and floating electrodes were fabricated by the successive deposition of palladium (Pd) and gold (Au) metals on the aligned CNT networks via photolithography and lift-off processes (Figure 1(c)). Gold metal was used to construct the electrodes due to its high electrical conductivity. Since the Schottky barrier height of a Pd-CNT contact is small, Pd layers were applied as a connector between the Au layers and the CNT networks to enhance the sensitivity of the nanodevices [32]. After that, the source and drain electrodes were insulated by an aluminum oxide (Al_2_O_3_) layer to restrict leakage of electrical currents (Figure 1(d)). This step was performed by using an atomic layer deposition system and an etching technique. After the fabrication process, the SCN devices were stored in a dry chamber for further experiments. Particularly, since the fabrication of our SCN devices was quite simple, the SCN devices were freshly prepared even before measurements.

### 2.3. AFM Imaging Procedure

The AFM imaging of a SCN was performed by using a commercial AFM system (MFP-3D, Asylum Research) in a tapping mode with a scan rate of 0.7 Hz.

### 2.4. Liquid Gating Effect Measurement of a SCN

A gold electrode was utilized to apply a liquid gate bias (*V*_*g*_) on a nanodevice through a Ca^2+^-free bath solution containing NaCl (140 mM), KCl (4 mM), MgCl_2_ (1 mM), D-glucose (10 mM), and HEPES (10 mM). The pH of the bath solution was adjusted to 7.4 with a NaOH solution. For liquid gating effect measurements, a gate bias was swept from −0.5 to 0.5 V while a source-drain bias was maintained at 0.1 V. The source-drain current of SCNs was measured using a semiconductor characterization system (Keithley, 4200, USA).

### 2.5. Electrophysiological Measurements by Using SCNs

For the electrophysiological monitoring of HeLa cells, a SCN device was assembled in a hand-made cell chamber. Before the electrophysiological measurements, the HeLa cells were washed three times with the Ca^2+^-free bath solution and incubated in it for further 60 minutes. And then, the HeLa cells were detached from the culture dish and transferred to the cell chamber. Subsequently, a HeLa cell was loaded onto the junction area of the SCN device assembled in the chamber by using a microcapillary (Figure 1(e)). To measure the conductance (G) of the SCN device, a voltage bias of 0.1 V was applied to the drain-source electrodes of the nanodevice while source-drain currents were recorded with a data acquisition system (National Instruments, NI-9215 (A)). The HeLa cell was stimulated by the addition of a histamine solution without Ca^2+^ into the chamber to give a final histamine concentration of 100 *µ*M.

For the study of the effects of chlorphenamine, cells were incubated with various concentrations of chlorphenamine (from 25 to 200 *µ*M) for further 15 minutes before the loading of the cells on a SCN was performed. And then, the cells were stimulated by histamine as in the above description.

## 3. Results and Discussion

### 3.1. Basic Characteristics of a SCN Device

**Figure 2:**
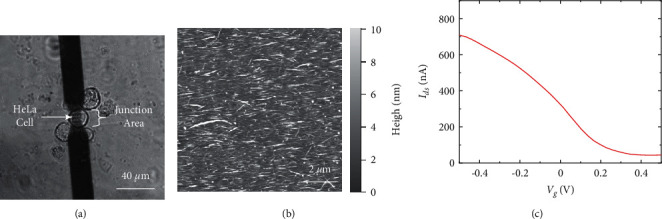
Basic characteristics of a SCN device. (a) Optical image showing a HeLa cell placed on a SCN device with three floating electrodes. (b) AFM topography image of the exposed area of a SCN device with aligned CNTs. (c) Liquid gating effect of a SCN device showing the decrease of a drain-source current *I*_*ds*_ along with the increase of a gate bias *V*_*g*_.

Figure 2(a) shows the optical image of a single HeLa cell placed on the junction area of a SCN device using a microcapillary. The image shows the dimensions of each floating electrode to be 2 *µ*m in width and 10 *µ*m in length. It was reported that the formation of floating electrodes could enhance the sensitivity of our SCN devices due to the number increase of Schottky barriers in the devices [33]. Moreover, the width of an exposed junction area is around 15 *µ*m, which fits the size of a HeLa cell. It indicates that our SCN device could be used to monitor electrophysiological responses at a single-cell level. Note that, in our work, HeLa cells were located in the junction area of a device, but they were not directly cultured on the device surfaces. This method allows us to exclude the binding of cell surface proteins to CNTs and maintain the primary characteristics of SCN devices. Furthermore, the measured cell could be easily removed from the device surface by mechanical shaking. An autopipette was utilized to shake a bath solution on the device, allowing us to withdraw the measured cells from the CNT junction area. Therefore, our nanodevice could be reused for other measurements with different cells, which allowed us to obtain results without suffering from errors due to device-to-device variations.

The surface topography image of a junction area between the electrodes of a SCN device was taken by atomic force microscopy (AFM) (Figure 2(b)). The image clearly shows the high alignment of CNT networks, which could increase the density of CNTs in the junction area. Also, the CNT alignment enables the SCN devices to enhance the conductivity due to the reduction of lateral CNT connections [34, 35]. Therefore, aligned CNT networks could significantly enhance the transconductance and sensitivity of nanodevices.

Figure 2(c) shows the gating effect curve of a SCN device obtained by applying a liquid gate bias through a gold electrode. The drain-source current through the device was recorded, while a bias voltage between the source and drain electrodes was maintained at 0.1 V. The curve expresses a decreasing drain-source current with the sweeping of a gate bias voltage from −0.5 to 0.5 V. This indicates that the semiconducting characteristics of SCN devices are similar to those of other classical p-type transistors [36, 37]. Moreover, the intense decrease of the drain-source current caused by a small gate bias change exhibits the high sensitivity of our device. Significantly, even though the device undergoes the harsh temperature and pressure conditions of fabrication procedures, it still expresses outstanding electrical characteristics. This clearly proves the stability of spin-coated CNT networks on a glass substrate.

### 3.2. Monitoring of Ca^2+^ Release from Intracellular Stores

**Figure 3:**
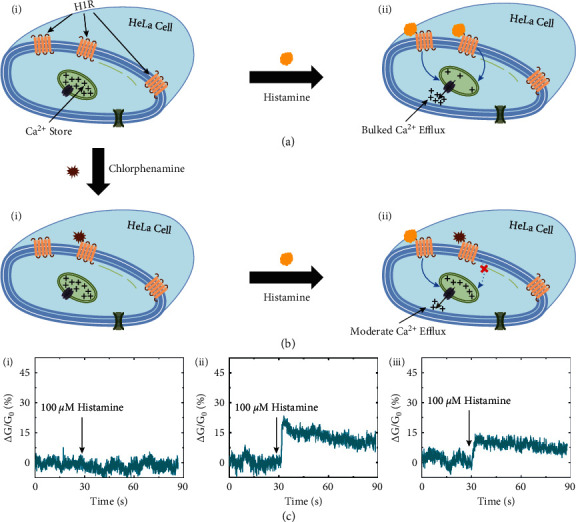
Real-time monitoring of the electrophysiological activities of a HeLa cell under histamine stimulation. (a) Schematic drawing depicting the mechanism of Ca^2+^ release from intracellular stores of HeLa cells stimulated by histamine: (i) resting state of a HeLa cell; (ii) stimulation of H1Rs by histamine resulting in the Ca^2+^ release from intracellular stores. (b) Schematic drawing depicting the effects of chlorphenamine on histamine-induced Ca^2+^ release from intracellular stores: (i) inactivation of H1Rs by chlorphenamine; (ii) reduction of histamine-induced Ca^2+^ release caused by a cell pretreatment with chlorphenamine. (c) Real-time monitoring of the conductance changes of a SCN device (i) without a HeLa cell, (ii) with a chlorphenamine nontreated HeLa cell, and (iii) with a chlorphenamine pretreated HeLa cell during the addition of 100 *μ*M histamine.

In nonexcitable cells such as HeLa cells, the agonist-induced Ca^2+^ release from intracellular stores has been reported to be a predominated mechanism for the regulation of cytosolic free Ca^2+^ concentrations [38–40].  Figure 3(a) depicts the mechanism of the Ca^2+^ release from the intracellular stores of HeLa cells under the stimulation of histamine. HeLa cells are known to possess H1Rs in their plasma membranes which can mediate the activation of Ca^2+^ stores via second messengers (Figure 3(a)-i) [40]. The binding of histamine to H1Rs forms IP_3,_ which acts as a second messenger causing the Ca^2+^ release from cytosolic stores (Figure 3(a)-ii). This Ca^2+^ release may cause the reduction of cellular membrane potential, which results in the accumulation of negative charges on the extracellular side of the plasma membrane [41]. Note that our SCN devices operate as p-type transistors, which means that the conductance of the SCN devices increases in the presence of negative charges in the interface between the cells and CNTs (Figure 2(c)). Therefore, the histamine-induced Ca^2+^ release can increase the conductance of SCN devices.

Figure 3(b) describes the effect mechanism of chlorphenamine on Ca^2+^ release from its store. H1Rs bound to chlorphenamine are transformed into inactive states, resulting in the downregulation of H1Rs activity under the stimulation of histamine (Figure 3(b)-i) [28, 29]. As a result, the release of Ca^2+^ from intracellular stores is degraded, which reduces the density of negative charges on the extracellular membrane (Figure 3(b)-ii). Thus, the effects of chlorphenamine on the activities of intracellular Ca^2+^ stores can be estimated by monitoring the conductance changes of the SCN devices.

The real-time relative conductance changes (ΔG/G_0_) of a SCN device without or with HeLa cells were monitored during the addition of 100 *μ*M histamine (Figure 3(c)). Here, ΔG is the difference between the instantaneous conductance value (G) and the initial conductance value (G_0_) of the device during measurements. Previous work demonstrated that when the histamine concentration was greater than or equal to 100 *μ*M, the responses of HeLa cells were the largest and similar to each other [42]. Therefore, the histamine concentration of 100 *μ*M was chosen to clearly observe the effects of various chlorphenamine concentrations on the electrophysiological responses of HeLa cells.  Figure 3(c)-i shows the response of the bare SCN device to the histamine addition. The data clearly present that there were no significant changes in the conductance of the SCN device without a cell during the addition of histamine. This implies that histamine solutions do not affect the conductance characteristics of the nanodevice. However, in the presence of a HeLa cell, the addition of histamine caused ∼19% relative conductance increase of the SCN device (Figure 3(c)-ii). This results from the activation of H1Rs in the HeLa cell membrane, which triggered Ca^2+^ release from its stores as well as the changes of membrane potential (Figure 3(a)). After that, the conductance of the SCN trended downwards, which was probably due to the repolarization of the cell membrane.

Figure 3(c)-iii shows the real-time electrophysiological responses of a HeLa cell pretreated with chlorphenamine to the stimulation of histamine monitored by using the same SCN device. Here, the HeLa cell was incubated in a bath solution containing 50 *μ*M chlorphenamine for 15 minutes before the cell was loaded onto the nanodevice. Then, the histamine stimulation was performed in a similar way to the untreated cell. After the addition of histamine solution, the relative conductance of the device increased by ∼10%, which was much lower than that observed in the untreated cell case (∼19%). This result confirms the antihistamine effects of chlorphenamine on HeLa cells as previous works [28, 29]. Moreover, these data clearly demonstrate the real-time monitoring ability of our SCN devices for the histamine-induced electrophysiological activities of HeLa cells. It also suggests that the effects of chlorphenamine on histamine-evoked Ca^2+^ release could be estimated by using the SCN device. Significantly, the single-cell-level monitoring capability of our method should be an interesting point for electrophysiological research.

### 3.3. Reusability of a SCN

**Figure 4:**
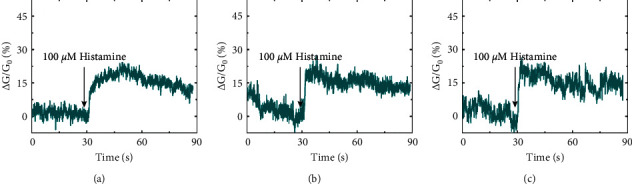
Repeated measurement of the real-time electrophysiological responses of individual HeLa cells under the simulation of 100 *μ*M histamine measured by using a single SCN device.

A single SCN device was utilized to perform three individual measurements with different HeLa cells under the stimulation of the same histamine solution (Figure 4). In this work, after each measurement, the HeLa cells were sucked out with a bath solution using an autopipette. The device was then rinsed with deionized water to recover the original surface of the SCN device. Since the cells did not adhere to the device surface, they could be easily washed out by shaking solution without any damage to the stability of CNT networks. At the beginning of Figure 4(b), the high ΔG/G_0_ values are probably caused by the diffusion of ions and compounds from the bath solution to a gap between the cell and CNT junction of the device. However, a histamine solution was added when the conductance of the SCN device was stable. This implies that the ΔG/G_0_ changes result from the responses of the HeLa cells to histamine. Significantly, the conductance changes of the SCN device before and after the addition of histamine in three experiments were quite similar to each other. This results from the preservation of device characteristics since, in our method, the HeLa cells do not need to be directly cultured on the device. The mean and relative standard deviation values of the relative conductance changes obtained from three measurements were calculated to be 19.39 and 0.81%, respectively. This clearly indicates the reusability and repeatability of our SCN devices. The signal-to-noise ratio (SNR) of real-time measurements by using semiconducting CNT transistors is a critical factor for the sensitivity and applicability of the transistors [43]. The obtained data clearly show a large SNR, enabling us to distinguish the responses of the SCN devices to the electrophysiological activities of HeLa cells. Current noninvasive techniques based on nanodevices have had difficulty in the quantitative monitoring of individual intracellular ion fluxes. It is because ion fluxes do not independently work off each other. However, these techniques could still investigate the effects of agents on the cellular electrophysiological activities. For example, our nanodevice could measure cell membrane potential alterations under histamine stimulation without any cell invasion as patch clamp techniques, which builds a simple and convenient method for electrophysiological research.

### 3.4. Quantitative Monitoring of Drug Effects by Using a SCN

**Figure 5:**
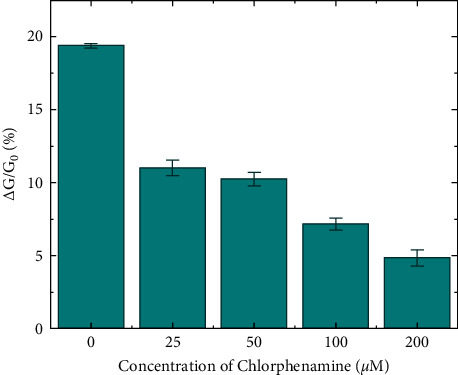
Reduction of histamine-induced Ca^2+^ release from the intracellular stores of HeLa cells pretreated with chlorphenamine at different concentrations in the range of 0–200 *μ*M. The responses of the HeLa cells were monitored by the measurement of the conductance changes of only a SCN device. Data are expressed as mean ± SD (standard deviation) (*n* = 3).

The electrophysiological response monitoring of HeLa cells pretreated with various concentrations of chlorphenamine to histamine stimulation was also carried out using only a SCN device. Here, HeLa cells were incubated with chlorphenamine solutions at a range of concentrations from 0 to 200 *µ*M. A 100 *µ*M histamine solution was used to activate Ca^2+^ release from intracellular stores of the pretreated cells.  Figure 5 exhibits that the conductance changes caused by the pretreated HeLa cells are smaller than those caused by the untreated cells. This confirms that chlorphenamine acted as an inverse agonist on the histamine-stimulated H1Rs of HeLa cells, which is consistent with previous reports [28, 29]. Furthermore, the result also shows the dose-dependent inhibition of chlorphenamine on the activities of H1Rs, which was previously studied on other cell lines [44]. For example, the conductance increase of the SCN device induced by the cell pretreated with a 25 *μ*M chlorphenamine solution is about 60% compared with that induced by the untreated cell. Note that we utilized the same device to monitor the effect of chlorphenamine on the histamine-induced Ca^2+^ release, indicating that our device could be applied for the quantitative real-time monitoring of the drug's effects without errors from the variation of device characteristics.

## 4. Conclusion

We demonstrate a method for the real-time monitoring of the effects of histamine on the intracellular Ca^2+^ release of a single cell. Here, a field-effect transistor was fabricated based on aligned semiconducting single-walled CNTs so as to measure the electrophysiological responses from HeLa cells under the stimulation of histamine. Our SCN device was also utilized to monitor the regulation of chlorphenamine on the activation of H1Rs induced by histamine. The pretreatment of the HeLa cells with chlorphenamine reduced Ca^2+^ release from intracellular stores as well as the expression of H1Rs. Considering that the electrophysiological responses of multiple individual cells could be measured by using a single SCN device, statistically meaningful measurements can be performed without errors from device-to-device variations. Significantly, the real-time monitoring capability of our method would promise various applications of nanodevices in electrophysiological studies and drug screening.

## Data Availability

The main data of the research were shown in the article. The detailed data can be available from the corresponding author upon request.
